# A seven-gene CpG-island methylation panel predicts breast cancer progression

**DOI:** 10.1186/s12885-015-1412-9

**Published:** 2015-05-19

**Authors:** Yan Li, Anatoliy A. Melnikov, Victor Levenson, Emanuela Guerra, Pasquale Simeone, Saverio Alberti, Youping Deng

**Affiliations:** 1 Rush University Medical Center, 653 W Congress Pkwy, Chicago, IL 60612 USA; 2 US Biomarkers, Inc, 29 Buckingham Ln., Buffalo Grove, IL 60089 USA; 3 Unit of Cancer Pathology, CeSI, ‘G. d’Annunzio’ University Foundation, Via L. Polacchi 11, 66100 Chieti, Italy; 4 Department of Neuroscience, Imaging and Clinical Sciences, Unit of Physiology and Physiopathology, ‘G. d’Annunzio’ University, Via dei Vestini, 66100 Chieti, Italy; 5 Currently at Center for Translational Research, Catholic Health Initiatives, Englewood, USA

**Keywords:** Breast cancer, DNA methylation, Microarray, Metastatic relapse, Prognostic indicators

## Abstract

**Background:**

DNA methylation regulates gene expression, through the inhibition/activation of gene transcription of methylated/unmethylated genes. Hence, DNA methylation profiling can capture pivotal features of gene expression in cancer tissues from patients at the time of diagnosis. In this work, we analyzed a breast cancer case series, to identify DNA methylation determinants of metastatic versus non-metastatic tumors.

**Methods:**

CpG-island methylation was evaluated on a 56-gene cancer-specific biomarker microarray in metastatic versus non-metastatic breast cancers in a multi-institutional case series of 123 breast cancer patients. Global statistical modeling and unsupervised hierarchical clustering were applied to identify a multi-gene binary classifier with high sensitivity and specificity. Network analysis was utilized to quantify the connectivity of the identified genes.

**Results:**

Seven genes (BRCA1, DAPK1, MSH2, CDKN2A, PGR, PRKCDBP, RANKL) were found informative for prognosis of metastatic diffusion and were used to calculate classifier accuracy versus the entire data-set. Individual-gene performances showed sensitivities of 63–79 %, 53–84 % specificities, positive predictive values of 59–83 % and negative predictive values of 63–80 %. When modelled together, these seven genes reached a sensitivity of 93 %, 100 % specificity, a positive predictive value of 100 % and a negative predictive value of 93 %, with high statistical power. Unsupervised hierarchical clustering independently confirmed these findings, in close agreement with the accuracy measurements. Network analyses indicated tight interrelationship between the identified genes, suggesting this to be a functionally-coordinated module, linked to breast cancer progression.

**Conclusions:**

Our findings identify CpG-island methylation profiles with deep impact on clinical outcome, paving the way for use as novel prognostic assays in clinical settings.

**Electronic supplementary material:**

The online version of this article (doi:10.1186/s12885-015-1412-9) contains supplementary material, which is available to authorized users.

## Background

Breast cancer predictive and prognostic procedures have a significant impact on current medical care. However, traditional prognostic parameters (lymph node diffusion, tumor size, grading, estrogen receptor expression) cannot adequately predict tumor relapse. As an example, 10–20 % of the patients with the best prognosis, i.e. with small size tumors, expressing estrogen receptors and without lymph node invasion, still experience relapse within 5 years [1, 2]. At the time of diagnosis, progressing cases cannot be distinguished from those that do not relapse by any conventional prognostic parameter. Therefore, effective markers, with better performance than traditional prognostic indicators, are urgently needed.

By merging biological insight and cluster analysis for experimental immunoistochemistry (IHC) parameters, we have previously succeeded in subgrouping breast cancers with distinct outcomes [3-5], and response to therapy [4, 6], indicating the clinical usefulness of such procedures. DNA methylation regulates gene expression, through the inhibition/activation of gene transcription of methylated/unmethylated genes, respectively [7, 8]. This largely occurs through methylation of CpG islands, most frequently in the promoter region of the genes [7, 9-11]. Broad hypomethylation with focal hypermethylation are frequently found in cancer [8, 12], thus affecting the expression of tumor suppressor genes, e.g. *TP53, DCC, SOCS2, DLEU7* [13-16], and favoring the mutation of oncogenes [17]. In turn, tumor suppressors have been shown to modulate DNA methylation levels, genome stability and DNA methylation-dependent gene amplification [18, 19], suggesting key interplays between alterations of DNA methylation and tumor progression. Indeed, DNA methylation-mediated loss of expression has been shown to cause functional ablation of hemizygous alleles at loss of heterozygosity (LOH) loci, encoding transcription factors (TF), e.g. *MOS, TTF-1* [20, 21], or proteins associated with DNA repair [22, 23], proteolytic processing [24], morphogenesis [25], control of cell cycle, signal transduction or apoptosis [26]. In breast cancer, CpG-island methylation was shown to inhibit *PTCH1* [27], *EFEMP1* [28] and *ESR1* [29] expression.

DNA methylation patterns can be assessed in formalin-fixed paraffin-embedded tissues (FFPE) tumor samples [30], allowing to profile gene expression regulatory mechanisms in tumors at the time of surgery, through methylation-sensitive restriction enzyme-analysis over a 56-gene cancer-specific biomarker microarray (MethDet-56) [31]. Long-term follow-up then permits to dissect correlations between DNA methylation profiles and biological outcome [32-35]. In this work we identified CpG-island methylation profiles of cancer biomarker regulatory regions, with a deep impact on prognostic determination in breast cancer, and the ability to distinguish cases with limited or nil risk for progression from those at high risk.

## Methods

### Breast cancer case series

A multi-institutional case series of breast cancer patients was collected from the University of Udine, the Venice and Rovigo hospitals, and Rush University (Additional file 1: Table S1). 123 breast cancer patients were analyzed; 19 cases showed metastases or metastatic relapse within 5 years from surgery (15.4 %) (Additional file 1: Table S1). Metastatic/relapsing cases were compared with patients that did not progress. Clinical and pathological data were obtained [36, 37] (Additional file 1: Table S1). Carcinoma grading was performed as described [38]. This project was approved by the Italian Ministry of Health (RicOncol RF-EMR-2006–361866), and by the Institutional Review Board of Rush University Medical Center. No written consent was needed for this study.

### DNA isolation

**Fig. 1:**
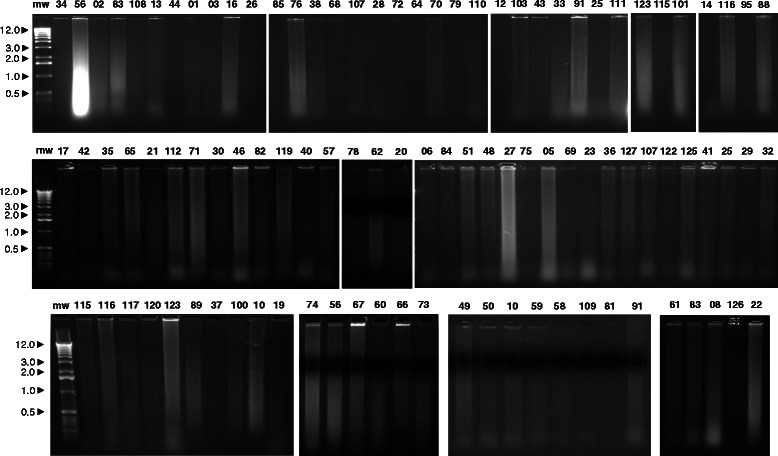
Breast cancer genomic DNA from the breast cancer case series. DNA was extracted from FFPE tissue samples as described and assessed by agarose gel/ethidium bromide electrophoresis. Numbers above each lane indicate individual patient’ cancer samples. mw: molecular weight markers in kilobases (left side of the panels)

Different procedures were compared for efficiency of DNA isolation from FFPE breast cancer samples from mastectomy or excision biopsy [31, 39, 40]. Samples were then processed for DNA extraction as described [31, 40]. Briefly, xylene deparaffination was followed by deproteinization with proteinase K in SDS-containing buffer at 56 °C. DNA was purified using DNAeasy Tissue kits (Qiagen). DNA was quantified using Hoechst 33258 or ethidium bromide fluorescence [41]. Agarose gel electrophoresis profiled DNA size distributions for sample quality assessment (Fig. 1).

### Microarray-mediated methylation assay

Bisulfite-based modification permits analysis of all cytosines in a sample; however, it leads to excessive fragmentation of DNA [42]. Affinity-based techniques require a substantial amount of starting sample, and their efficiency depends on the density of methylation marks within each specific fragment [43]. Restriction enzyme-dependent methods are more flexible for analysis of small samples, and are focused on assessing methylation of selected restriction sites [8, 31]. As DNA from human cancer samples is a limiting factor, we utilized previously developed procedures of methylation-sensitive restriction enzyme-cleavage [31].

**Fig. 2:**
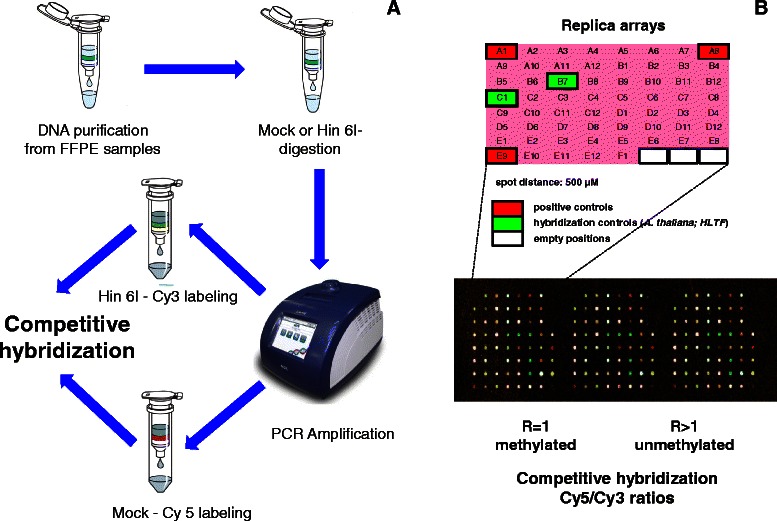
Microarray-mediated methylation assay. (**a**) Flow chart of the methylation-sensitive restriction enzyme-based MethDet-56 approach. (**b**) Subarray layout (top) and representative whole-slide hybridization (bottom)

For each patient, DNA methylation was tested over a 56-gene cancer-specific biomarker microarray as previously described [44] (a flowchart is provided in Fig. 2a). Briefly, each DNA sample was split into two aliquots; one of these was digested with Hin6I, while the second one was mock-digested. Both samples were amplified by nested PCR, and 5-aminoallyl dUTP (Biotium Inc.) was added to the second amplification run. Products of the Hin6I-digested DNA were then labeled with Cy3, while products of the mock-digested DNA were labeled with Cy5. Labeled DNAs were mixed and competitively hybridized to DNA microarrays. Slides were scanned using a GenePix 4000B Microarray Scanner (Molecular Devices). Intensity of fluorescence was determined using the GenePix Pro 6.1.0.2 software. Raw GenePix data were imported as the ratio of signal intensity of Control hybridization and Test hybridization for each spot, and processed using Agilent Genespring 12.5, with lowess normalization and base 2 log transformation. Microarray data are available in the ArrayExpress database [45] under accession number E-MTAB-3153.

### Data processing

Each microarray contained three identical subarrays of 64 (8 × 8) spots [31] (Fig. 2b). Gene CpG-island probes corresponded to 56 spots; three spots contained positive control DNAs and 2 spots contained hybridization control DNAs, to quantify specific versus nonspecific binding; three empty areas were used to quantify background intensity. A multi-step filtering was applied as follows: spots were removed from analysis if signals were <2 times the average of the control spots, as calculated for each slide over the three subarrays. Next, data that had less than two informative spots across the three subarrays were removed. Genes with missing data in more than 25 % of the samples were removed from the analysis. For continuous-variable approaches, the mean of the ratios was calculated for each gene of every sample. The methylation ratio (Cy5/Cy3) was then calculated, and the methylation status of each CpG island was categorized as either methylated or unmethylated.

### Statistical analysis

The initial lowess-normalized, log-transformed ratio data were grouped according to breast cancer progression status. Log-transformed data distributions were shown to follow a normal distribution. Hence, the means of values from the tumors that progressed were compared to the corresponding measurement from non-progressing tumors by independent sample student’s *t*-test of fold changes (FC). Cutoffs of absolute FC ≥2, p < 0.05 were used to filter-out genes that were not highly differentially methylated. The remaining genes were submitted to feature selection models utilizing JMP Genomics algorithms, with 5-fold, ten-runs cross-validation. The models utilized were discriminant analysis, general linear model selection, k-nearest neighbors, logistic regression, partial least squares (PLS) and partition trees. Based on the highest average area (AUC) under the receiver-operating-characteristic curve (ROC) value across ten runs, a model was chosen as the optimal binary classifier. That model was then used to select the genes with greatest effect on the classifier results. To confirm these results, individual-gene PLS analysis was performed using R’s pls package, and statistical power computations, as previously described [46, 47]. Hierarchical clustering using euclidean distance and centroid linkage were performed with Genespring. Statistical power was computed for each individual gene and for the averaged 7-gene panel using PASS 12.0 with a *t*-test model and no assumption of equal variance [46, 47]. A significance level (alpha) of ≥0.05 and a threshold of ≥80 % statistical power were adopted as analytical thresholds.

### Network analysis

Signaling hubs and connectivity networks were obtained using Ingenuity Pathway analysis [48] and STRING 9.1 [49] software. To increase specificity, the IPA analysis was confined to molecules and/or relationships observed in breast tissues and cell lines. STRING parameters included: Active Prediction Methods: Experiments ~ Databases ~ Textmining; required high-confidence (0.700); no more than ten interactors.

## Results

### CpG-island methylation analysis

DNA extraction was performed on FFPE breast cancer samples from 123 patients from a multi-institutional case serie with a minimum follow-up of 5 years. (Additional file 1: Table S1). Ethidium bromide gel electrophoresis (Fig. 1) and amplification of *RAS* and *TP53* exons (manuscript in preparation) benchmarked DNAs as viable for additional DNA methylation analysis. Relapsing cases were extracted from the registry and matched with non relapsing patients on the basis of clinico-pathological data (tumor diameter, pathological stage, tumor histotype, age, hormone receptors and grading). DNA methylation of transcription-regulatory regions in the selected case-control group was analyzed through methylation-sensitive restriction enzyme-cleavage, followed by PCR amplification and competitive hybridization of fluorescence-labelled PCR products on custom DNA microarrays containing CpG-island gene-probes for 56 cancer specific biomarkers, as described [31]. Samples were analyzed through triplicate spot arrays (eight by eight), each one containing three positive hybridization controls, two negative controls (*A. thaliana* and *HLTF*) and thre empty spots, to measure background fluorescence (Fig. 2). Hybridization raw data are available as indicated in Methods [45]. Microarray fluorescence measurements were acquired as test (Hin6I-digested DNA labeled with Cy3) versus control hybridization intensity (mock-digested DNA labeled with Cy5). Background fluorescence was subtracted, and Cy3/Cy5 intensity ratios were obtained for each spot. Intensity ratios were lowess normalized against global signal intensity of the array, and transformed as base 2 logs. For continuous-variable approaches, means of ratios were obtained for each gene analyzed. Spot signals were filtered according to absolute signal intensity (threshold of ≥2 times versus control spots), viable spots numbers (≥2) and missing data (spot series with missing data in ≥25 % of the samples were discarded).

### Gene CpG-island methylation ranking

Filtered methylation ratios (Cy5/Cy3) were utilized to define the methylation status of each CpG island. These were categorized as either methylated or unmethylated, using cutoffs of absolute FC ≥2, p <0.05. Normalized fluorescence ratios/differential methylation categorization were then assigned to the relapsing or non-relapsing patients groups. A first comparison between the means of the values from each group extracted 21 genes, that met the cutoff for significant differential methylation (Table 1). PLS K nearest-neighbors with a radial basis machine was applied to obtain a first ranking of significantly differentially-methylated genes (Additional file 2: Table S2). This identified *DAPK1*, *MDGI*, *BRCA1*, *P15*, *PGK1*, *PGR*, *SYK*, *THBS1*, *14-3-3σ*, *APAF1*, *CALCA* and *CCND2* as the highest differentially methylated genes between progressing and non-progressing breast cancers. This group of genes contained controllers of cell proliferation (*P15, CCND2, PGR*) and apoptosis (*APAF1, DAPK1*), p53 interactors (*THBS1, DAPK1*), signaling kinases (*SYK, PGK1, DAPK1*), drivers of tumor development (*BRCA1, 14-3-3* [50]), suggesting direct relevance of differentially-methylated/regulated genes for breast cancer development or progression.

**Table 1:** Gene CpG-island methylation profiles associated with metastatic relapse

ID^a^	*P-value*	Fold change (absolute)
BRCA1	1.53E-05	5.52
CALCA	1.96E-04	1.66
CASP8	3.54E-03	1.82
CCND2	2.81E-04	1.62
DAPK1	1.13E-06	12.37
EDNRB	2.00E-04	2.42
FHIT	6.95E-03	1.79
ICAM1	1.60E-04	2.06
MCTS1	3.36E-02	1.56
FABP3	3.04E-04	2.78
DNAJC15	2.03E-03	3.01
MSH2	3.10E-05	13.66
MYOD1	1.40E-04	1.74
CDKN2A	1.29E-02	1.83
PAX5	6.82E-03	1.77
PGK1	1.77E-03	1.79
PGR	2.54E-03	2.9
RARB	1.39E-04	2.05
PRKCDBP	7.68E-04	3.63
THBS1	1.26E-03	2.08
RANKL	1.10E-02	2.14

^a^: Filtered gene list, with cutoff fold change ≥1.5, *p*-value <0.05. PLS-selected genes are in bold

### Marker-gene profiling

**Fig. 3:**
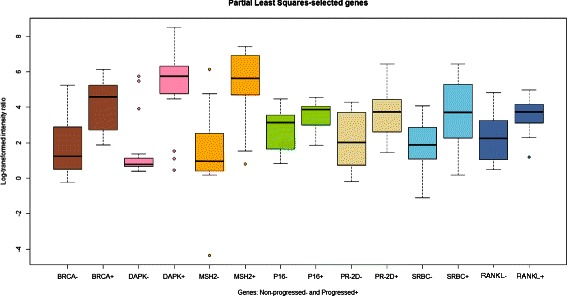
Boxplot of promoter methylation levels - PLS analysis-selected genes. Samples are paired, gene-color coded, and compare relapsed (+) versus non relapsed (−) cases

**Fig. 4:**
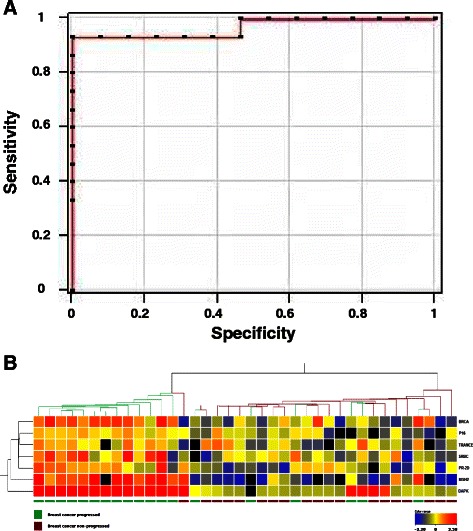
The 7-gene classifier. (**a**) ROC curve for all the PLS-selected genes. Highest AUC value across ten runs, for optimal binary classifier model. (**b**) Hierarchical clustering with euclidean distance. Sample colors represent deviation from the median (black is missing datapoint). Green bars indicate cancer-progressing samples, brown bars correspond to non-progressing samples. Notably, tight clustering of 13/19 relapsing cases was observed on one branch (left side), while 18/19 non-relapsing cases were clustered on the second branch (right side)

These findings led us to further refine our breast cancer prognostic model, through procedures of best-model fitting of differentially methylated gene profiles. The 21 genes that had been previously filtered (Table 1) were thus modelled using discriminant analysis, general linear model selection, k-nearest neighbors, logistic regression, PLS and partition trees. Model’ performances were evaluated on the basis of AUC values across five-fold ten-runs cross-validation. The PLS model generated the highest AUC (mean AUC ≈ 0.8) and was chosen as the best binary classifier of progressing versus non-progressing breast cancers. Genes selected by the PLS model were *BRCA1, DAPK1, MSH2, CDKN2A/P16, PGR, PRKCDBP/SRBC, RANKL/TNFSF11/TRANCE* (Fig. 3). These seven genes were shown to provide the maximal overall contribution to AUC measurements (Fig. 4a, Table 1), i.e. the greatest impact on the classifier, across all ten comparison runs (Fig. 4a, Table 1).

### Measuring the global accuracy of prognostic gene markers

We then went on to utilize model-fitting marker genes to generate accuracy measurements for binary classification of breast cancer relapse risk. The seven genes selected by PLS were subjected to whole-model fit using the entire data-set. Individual-gene data were then analyzed using R statistics. Individual-gene analyses highlighted sensitivities of 63–79 %, with 53–84 % specificity, positive predictive value of 59–83 % and a negative predictive value of 63–80 %. Modeling all seven genes together then allowed to reach remarkable sensitivity (93 %) and specificity (100 %), a positive predictive value of 100 % and a negative predictive value of 93 %. These findings indicated that while individual genes had effectiveness at classification, global analyses were much more proficient (Fig. 4a, Table 2). Notably, the ROC curve for all PLS-selected genes together had an AUC = 0.9643 (Fig. 4a).

**Table 2:** Accuracy rates of individual selected genes and aggregated models^a^

Model	Sensitivity	Specificity	Positive predictive value	Negative predictive value	Area under the ROC curve
All 7 genes^b^	0.93	1.00	1.00	0.93	0.97
BRCA1^c^	0.74	0.74	0.74	0.74	0.87
DAPK1^c^	0.79	0.84	0.83	0.80	0.89
MSH2^c^	0.74	0.84	0.83	0.76	0.89
CDKN2A^c^	0.68	0.53	0.59	0.63	0.76
PGR^c^	0.74	0.58	0.64	0.69	0.77
PRKCDBP^c^	0.63	0.68	0.67	0.65	0.80
RANKL^c^	0.68	0.68	0.68	0.68	0.75

^a^:comprehensive accuracy rates and individual parameters are listed

^b^: global contribution of all selected genes was assessed using JMP Genomics software

^c^: individual gene contributions were assessed using R statistics

### Statistical power of the CpG-island methylation analysis

Statistical power analysis indicated that the methylation patterns of five of the seven best predictors, i.e. *BRCA1*, *DAPK1*, *MSH2*, *PGR*, *PRKCDBP*, possessed a high discriminating power of relapsing cancer cases versus non-relapsing controls (Additional file 2: Table S3). *CDKN2A* and *RANKL* showed trends toward the high threshold and contributed non-redundantly to prediction when combined in the 7-gene set. Remarkably, the power of the 7-gene set was 1 (Table 3), strongly supporting the combined use of the seven gene markers.

**Table 3:** Statistical power analysis of the 7-gene classifier

ID	mean diff	stdev BCR	stdev BCS	statistical power	Gene Name
Average of 7	2.112189	1.16809	0.708074	1	*BRCA1*, *DAPK1*, *MSH2*, *CDKN2A*, *PGR*, *PRKCDBP*, *RANKL*

### Hierarchical clustering

Unsupervised hierarchical clustering of the tumor samples, based on the 7-gene classifier, was performed to independently assess the prognostic-association performance above. Hierarchical clustering demonstrated close agreement with the accuracy measurements. Of interest, tight clustering of 13 relapsing cases was oberved. At variance, 6 other progressing cases appeared broadly distributed among tumors with benign outcome (Fig. 4b), consistent with a distinct heterogeneity in biological trajectories to metastatization (manuscript in preparation).

### Gene pathways associated to prognostic determinants

Pathway analysis was performed on differentially-methylated genes (Additional file 2: Table S4; Additional file 3, 4: Figures. S1, S2). Most genes/gene relationships were found to map over key cancer and cell-signaling biological pathways (Additional files 3, 4: Figures S1B, S2). Ingenuity pathway analysis (IPA) highlighted a statistically significant network of interactions (score = 28, Fisher’s exact test p = 1x10^−28^) (Additional file 3: Figure S1A). 14 of the differentially methylated genes appeared closely connected, suggesting a functionally-relevant signaling module. This module converged on three major hubs: *ERBB2*, *PRG* and *BRCA1* (Additional file 3: Figure S1A). This analysis revealed the p53 signaling network as the most relevant one (*p* = 0.0000091); this encompassed the *CDKN2A, CCND2, THBS1* and *BRCA1* genes (Additional file 2: Table S4). These findings were independently validated by STRING network analysis (Additional file 4: Figure S2A) (p53 signaling network, KEGG entry: map04115, p-value = 6.17 E-22). Molecular Mechanisms of Cancer (IPA p = 0.000977) (Additional file 2: Table S4; Additional file 3: Figure S1B) and Pathways in cancer (KEGG entry: map05200 STRING p = 8.72 E-13) (Additional file 4: Figure S2B) appeared as additional relevant canonical network. Of interest, gene networks appeared related to broadly different cancer histotypes (Additional file 2: Table S4), suggesting a wide significance of this cancer-associated module, and potential relevance also in histotypes other than breast cancer.

## Discussion

In this work, we have identified a gene methylation panel for binary classification of breast cancer progression. Utilizing IHC parameters we had previously succeeded in subgrouping breast cancers [3-5] for prognostic and therapeutic use [6]. Profiles of DNA methylation/regulation of expression of pivotal cancer drivers [51] were expected to provide additional valuable information, and to critically complement current prognostic procedures. Hence, we went on to identify gene methylation profiles that could bear significant value for prognostic determination [31].

We first identified gene markers whose methylation patterns differed between progressing and non-progressing breast tumors. PLS k-nearest neigbors radial basis machine identified *DAPK1*, *MDGI*, *BRCA1*, *P15*, *PGK1*, *PGR*, *SYK*, *THBS1*, *14-3-3σ*, *APAF1*, *CALCA* and *CCND2*, as differentially methylated genes between progressing and non-progressing cancers. These genes included cell cycle regulators, signaling kinases, cytoplasmic scaffold/regulatory molecules and p53 interactors, suggesting direct relevance for breast tumor progression.

Hence, we went on to further refine our breast cancer prognostic model, through procedures of best-model fitting of differentially methylated gene profiles. PLS models were shown to provide the highest AUC and were chosen as the best binary classifiers of progressing versus non-progressing breast cancers. The genes that contributed most to a PLS binary classification model were *BRCA1*, *DAPK1*, *MSH2*, *CDKN2A*, *PGR*, *PRKCDBP*, *RANKL*. Individual-genes assessments showed 63–79 % sensitivity, 53–84 % specificity, positive predictive values of 59–83 % and negative predictive values of 63–80 %. A 5-fold cross-validation in selected models and PLS analysis through distinct procedures (JMP Genomics, Genespring and R) were used to assess gene clusters versus individual genes. Remarkably, when modelled together, the seven genes reached a sensitivity of 93 %, with 100 % specificity, a positive predictive value of 100 % and a negative predictive value of 93 %. The 97 % estimates for the AUC of the 7-gene panel model supported it as a reliable predictor of breast cancer progression, as did the normality of the log-transformed data distributions. Statistical power analysis supported the strength of our analytical strategies. The majority of the predictors (i.e. *BRCA1*, *DAPK1*, *MSH2*, *PGR*, *PRKCDBP*) demonstrated high statistical power, with a threshold of ≥80 % and an alpha significance level of 0.05. *CDKN2A* and *RANKL* were close to high statistical power thresholds and were shown to provide non-redundant information to prognosis when combined with *BRCA1*, *DAPK1*, *MSH2*, *PGR* and *PRKCDBP*. To assess the overall statistical power of the 7-gene set, an average for the seven prognostic genes was computed for each individual sample. Then, the power calculation was performed, as based on the distance between the mean of relapsing cancer samples versus that on non-relapsing cases. A remarkable power of 1 was obtained, strongly supporting the efficiency of 7-genes panel. Unsupervised hierarchical clustering of the tumor samples, demonstrated close agreement with the accuracy measurements. Of note, tight clustering of 13 relapsing cases was oberved, whereas 6 additional cases distributed among tumors with favourable outcome. These findings suggested heterogeneity in the biological paths that are followed to reach pro-metastatic states, in spite of the sharing of candidate causal genes.

Our model predicted that promoter DNA methylation, with subsequent transcriptional inactivation of the 7-gene set would be detrimental and associated with tumor progression. Individual genes findings fully supported this model.

BRCA1 is a tumor suppressor gene [52, 53] involved in DNA repair, cell cycle checkpoint control, and maintenance of genomic stability [54]. Germline mutations in BRCA1 predispose women to breast and ovarian cancers [55], with a 50–85 % lifetime risk of developing breast cancer [56]. Promoter hypermethylation was shown to cause loss of BRCA1 expression both in sporadic ovarian cancer [57] and in hereditary ovarian carcinomas [58]. Promoter methylation was detected in 31 % of carcinomas but in none of the benign or borderline tumors [59]. Levels of methylation in ovarian tumors quantitatively correlated with decreased BRCA1 expression [60, 61]. Hypermethylation of BRCA1 was detected at a significantly higher frequency in serous carcinomas than in tumors of the other histological types [62], with earlier onset of high-grade serous ovarian cancer. BRCA1 promoter methylation was frequently found in triple negative breast cancers and identified a significant fraction of patients with poor outcomes [63]. Notably, promoter methylation of BRCA1 was also found in 46 % of pancreatic neoplasms [64] suggesting a broader impact of this alteration, beyond ovarian and breast cancers.

Death-associated protein kinase (*DAPK*) is a pro-apoptotic determinant which is dysregulated in a wide variety of cancers [65]. Hypermethylation of *DAPK1* is the most frequent molecular alteration identified in immunodeficiency-related lymphomas [66], and was detected in almost all cases of chronic lymphocytic leukemia [67]. Hypermethylation patterns of *DAPK* were found in head and neck cancers [68], bladder tumors [69], and brain metastases of solid tumors [70], and were associated with poor outcome. The DNA methyltransferase inhibitor 5-Azacytidine (5-Aza) was shown to induce promoter demethylation and to restore mRNA expressions of *DAPK* in osteosarcoma cells [71], confirming DNA methylation as a determinant of transcriptional inactivation of this gene.

*PGR* (progesterone receptor) is a member of the steroid receptor family and mediates the gene transcription regulatory effects of progesterone. The *PGR* status yields prognostic information in patients with node-negative breast cancer [72]. Lack of expression of *PGR*, together with loss of estrogen receptors and of Her-2/neu, identifies ‘triple negative’ breast cancers, which are an aggressive, poor-outcome breast cancer subgroup [4]. *PGR* was inactivated by promoter methylation in tamoxifen-resistant breast cancer cells. Following promoter demethylation with 5-Aza, the co-addition of oestradiol (E2) restored gene expression, and inhibited cell proliferation [73]. *PGRß* was found hypermethylated in 56 % of melanoma cell lines [74], and in acute myeloid leukemias [75].

Protein kinase C δ-binding protein is encoded by the *PRKCDBP* (*SRBC*) gene. Frequent epigenetic or mutational inactivation of *PRKCDBP* was observed in sporadic breast, lung, ovarian, and other types of adult cancers as well as childhood tumors [76]. The expression of the PRKCDBP protein was down-regulated in about 70 % of breast, lung, and ovarian cancer cell lines, whereas a strong expression of the protein is detected in normal mammary and lung epithelial cells [76]. *PRKCDBP* is frequently shut-down in glioblastoma multiforme [77] and in colorectal cancer [78] by promoter hypermethylation [79]. *PRKCDBP* methylation in neuroblastoma was associated with unfavourable outcome [80]. *PRKCDBP* is a proapoptotic tumor suppressor which is activated by NF-κB in response to TNFα, suggesting that *PRKCDBP* inactivation may contribute to tumor progression by reducing cellular sensitivity to TNFα. Loss of expression of the PRKCDBP protein was associated to hypermethylation in non-small-cell lung cancers and breast cancer cells; re-expression was observed after treatment with 5-Aza [76, 81].

p16 is a cyclin-dependent kinase inhibitor and a tumor suppressor protein. Loss of the corresponding locus (*CDKN2A*) is among the most frequent cytogenetic alteration events in human cancer [82]. The frequency of inactivation of p16 by DNA methylation is even higher than that by genetic changes in many cancers, e.g. in gastric carcinomas (32–42 % of cases), where this is an early event and is associated with poor clinical outcome [83]. Correspondingly, p16 methylation is detected in precancerous and inflammatory lesions of colon, lung, liver, oral cavity [84], and is associated with malignant progression [85, 86]. p16 methylation is associated with lower overall survival and disease-free survival in non-small cell lung cancer patients [87], melanomas [88] and paragangliomas [85]. p16 is frequently methylated/inactivated in haematopoietic malignancies, such as acute lymphoid leukaemia (ALL), lymphomas and multiple myeloma [89]. 5-Aza was shown to restore gene transcription of hypermethylated *CDKN2A* genes [90]. Taken together, these findings have led to the FDA approval of 5-Aza for treatment of patients with myelodysplastic syndromes [89].

MSH2 is a tumor suppressor protein involved in DNA repair, e.g. base excision, and transcription-coupled homologous recombination [91-93]. Heterozygous LOH germline mutations of *MSH2* are causal factors of the Lynch syndrome (hereditary non-polyposis colorectal cancer, HNPCC) [94]. Heritable transmission of propensity to *MSH2* methylation in a family with HNPCC has been reported [95]. Aberrant DNA methylation and epigenetic inactivation of *MSH2* play a role in the development of ALL, through induction of cell growth and survival [96]. CpG island methylation in *MSH2* associates with carcinogenesis in colorectal carcinomas presenting with a conventional adenoma-carcinoma sequence. Therefore, the detection of *MSH2* methylation may have clinical significance in the evaluation of colon cancer patients and in a precision-medicine management of the disease [97].

*RANKL* (*TNFSF11*, *TRANCE*) is a TNF family member, and, together with its receptor RANK, is a key regulator of cell survival. The RANKL/RANK system is modulated by osteoprotegerin (OPG) which binds to RANKL and prevents its interaction with RANK. RANKL activates Akt1 through a signaling complex involving Src and TRAF6 [98]. RANK is found expressed on cancer cell lines and breast cancer cells in patients [99]. The RANK/RANKL signaling plays an essential role in progestin-induced breast cancer development [100] and stimulates breast cancer metastasis [101]. Corresponding, RANKL triggers the migration of cancer and melanoma cells that express the RANK receptor [99]. The methylation status of both RANKL and OPG quantitatively controls their levels of expression [102]. Consistent, RANKL expression in myeloma cells was shown to be driven by TNFα-induced gene demethylation [103]. Thus, RANKL and OPG act as a cancer/metastasis control module, whose balance is determined by epigenetic regulation.

Network analysis was used to identify functional interrelationship across the tumor progression predictors identified. Notably, most of these prognostic genes and their direct interactors in the network were found to map over key cancer cell-signaling pathways. These close relationships suggested the existence of a functional signaling module, which converged on the *ERBB2*, *PRG* and *BRCA1* hubs. Remarkably, this network module appeared most related to the p53 signaling pathway (*p*-value = 0.0000091). *TP53* is the most frequently mutated gene in cancer [104-106] and *TP53* mutations are specifically associated to tumor subgroups with distinct biological features, particularly in breast cancer [4, 5, 33, 107]. Notably, though, the 7-gene-driven network was shown to be active also in cancer histotypes other than breast (non-small cell lung cancer, bladder, ovary), suggesting an even broader relevance for tumor progression.

## Conclusions

Our findings identify CpG-island methylation profiles of seven genes, i.e. *BRCA1*, *DAPK1*, *MSH2*, *CDKN2A*, *PGR*, *PRKCDBP*, *RANKL,* as having a deep impact on clinical outcome. Our findings candidate the 7-gene methylation profile as a tool for quantifying the risk of relapse of breast cancers, paving the way for use as a novel prognostic assay in clinical settings.

## Additional files

Additional file 1: Table S1. Pathological and clinical parameters of the breast cancer case series.
Additional file 2: Table S2. PLS K nearest-neigbors, radial basis machine ranked genes. Table S3: Statistical power analysis of single-gene DNA-methylation patterns. Table S4: Pathway analyses.
Additional file 3: Figure S1. Gene-protein interaction network - IPA analysis. (**A**) Network display. Red nodes are directly linked to input genes (Tables 1, 2); white nodes indicate higher iteration/depth. Edges are predicted functional links, and are indicated by arrows. Continuous lines: direct interactions; dashed lines: indirect interactions. (**B**) Overlay on the Canonical cancer signaling Pathway. Proteins directly linked to input genes (Tables 1, 2) are highlighted in red. Nuclear or cytoplasmic localization is indicated. Relationship with apoptotic pathways is detailed.
Additional file 4: Figure S2. Gene-protein interaction network - STRING analysis. Networks including input proteins and protein bridges. (**A**) p53 signaling pathway (KEGG entry: map04115 *p* = 6.17 E^−22^) proteins are highlighted in blue. (**B**) Pathways in cancer (KEGG entry: map05200 p = 8.72 E^−13^) proteins are highlighted in red; additional cancer-relevant proteins are highlighted in pink.

## Abbreviations

ALL: Acute lymphoid leukaemia
AUC: Area under the curve
FFPE: Formalin-fixed paraffin-embedded
IHC: Immunohistochemistry
IPA: Ingenuity pathway analysis
KEGG: Kyoto Encyclopedia of Genes and Genomes
LOH: Loss of heterozygosity
PCR: Polymerase chain reaction
PLS: Partial least squares
ROC: Receiver operating characteristic
STRING: Search Tool for the Retrieval of Interacting Genes/Proteins
