# Supplementary material for: A seven-gene CpG-island methylation panel predicts breast cancer progression
Source: BMC Cancer. 2015 May 19;15:417. doi: 10.1186/s12885-015-1412-9 (PMC4438505; doi:10.1186/s12885-015-1412-9)
Supplement: Additional file 2: Table S2. — PLS K nearest-neigbors, radial basis machine ranked genes. Table S3: Statistical power analysis of single-gene DNA-methylation patterns. Table S4: Pathway analyses. [file 12885_2015_1412_MOESM2_ESM.doc]

# **Table S2: PLS K nearest-neigbors, radial basis machine ranked genes.**

| **Rank** | **Selected genes**^a^ |
| --- | --- |
| **1** | **DAPK1_1** |
| **2** | **MDGI_1** |
| **3** | **BRCA1_0** |
| **4** | **DAPK1_0** |
| **5** | **P15_1** |
| **6** | **PGK1_1** |
| **7** | **PGR_0** |
| **8** | **SYK_1** |
| **9** | **THBS1_0** |
| **10** | **14-3-3_1** |
| **11** | **ACTIN_1** |
| **12** | **APAF1_1** |
| **13** | **BRCA1_1** |
| **14** | **CALCA_1** |
| **15** | **CCND2_1** |
| **16** | **EDNRB_0** |
| **17** | **EDNRB_1** |
| **18** | **EP300_1** |
| **19** | **ERaA_1** |
| **20** | **ERaB_1** |
| **21** | **E-CAD_1** |
| **22** | **FHIT_1** |
| **23** | **GPC3_1** |
| **24** | **GR_1** |
| **25** | **GSTP1_1** |
| **26** | **HIC_1** |
| **27** | **HIN1_1** |
| **28** | **HMLH1_1** |
| **29** | **HSHLTF1_1** |
| **30** | **MCJ_0** |
| **31** | **MCJ_1** |
| **32** | **MDGI_0** |
| **33** | **MGMT_0** |
| **34** | **MSH2_0** |
| **35** | **MYF3_1** |
| **36** | **CDKN2A_0** |
| **37** | **P21_1** |
| **38** | **P27_1** |
| **39** | **P57_1** |
| **40** | **P73_1** |
| **41** | **PAX5_1** |
| **42** | **PGK1_0** |
| **43** | **PR_2D_0** |
| **44** | **RAR_1** |
| **45** | **RASS_1** |
| **46** | **RB1_1** |
| **47** | **RFC_1** |
| **48** | **RIZ_1** |
| **49** | **RPL15_1** |
| **50** | **S100_1** |
| **51** | **SOCS_1** |
| **52** | **SRBC_0** |
| **53** | **TES_1** |
| **54** | **RANKL_0** |
| **55** | **UPA_1** |
| **56** | **VHL_0** |

^a^:_1 and _0 indicate independent data assessments.

**Table S3. Statistical power analysis of single-gene DNA-methylation patterns**

| **ID** | **mean diff** | **stdev BCR** | **stdev BCS** | **statistical power** |
| --- | --- | --- | --- | --- |
| *14-3-3* | 0.732969262 | 2.052538855 | 2.952596907 | 0.13852 |
| *ACTIN* | 0.02120974 | 0.059474826 | 0.015236178 | 0.30019 |
| *APAF-1* | 0.013072969 | 0.02345244 | 0.018891562 | 0.45208 |
| *BRCA1* | 2.465830461 | 1.435531038 | 1.603266998 | 0.99811 |
| *CALC* | 0.735170986 | 0.610458459 | 0.457330212 | 0.98289 |
| *CASP 8* | 0.863180475 | 0.654473218 | 0.982125906 | 0.87055 |
| *CYC D2* | 0.699606443 | 0.678174788 | 0.337950871 | 0.97222 |
| *DAPK* | 3.628649249 | 2.102996704 | 1.638051095 | 0.99993 |
| *E-CADR* | 0.575162596 | 0.497907683 | 0.327621561 | 0.98272 |
| *EDNRB* | 1.276812449 | 1.128536008 | 0.730274129 | 0.97973 |
| *EP 300* | 0.222505562 | 0.381596077 | 0.417401187 | 0.38563 |
| *ERaA* | 0.33879209 | 0.409264336 | 0.429052211 | 0.67853 |
| *ERaB* | 0.02715477 | 0.795816577 | 0.885356648 | 0.05107 |
| *FAS* | 0.109627306 | 1.539909509 | 2.311637048 | 0.05319 |
| *FHIT* | 0.835965053 | 1.136583287 | 0.572458401 | 0.78774 |
| *GPC3* | 0.806868364 | 1.359465083 | 1.248433867 | 0.45797 |
| *GR* | 0.172562657 | 0.330549819 | 0.395004114 | 0.29508 |
| *GSTP* | 0.005857217 | 0.020997285 | 0.015570808 | 0.15774 |
| *HIC* | 0.753867155 | 1.69060317 | 1.323921558 | 0.31848 |
| *HIN* | 0.088212547 | 0.237176471 | 0.016982289 | 0.33457 |
| *HMLH1* | 0.02722384 | 0.675504302 | 0.454536546 | 0.05229 |
| *HSHLTF1* | 0.182993122 | 0.388169827 | 0.492452567 | 0.23538 |
| *ICAM1* | 1.043246134 | 0.884664902 | 0.572850544 | 0.98668 |
| *MCT1* | 0.640195754 | 1.07041392 | 0.670676002 | 0.57095 |
| *MDGI* | 1.474707693 | 1.307698127 | 0.903597323 | 0.975 |
| *MGMT* | 0.691866354 | 1.312407226 | 1.022798739 | 0.42133 |
| *MCJ* | 1.588938087 | 1.571329965 | 1.264486291 | 0.9157 |
| *MSH2* | 3.772050327 | 2.103426229 | 2.343504839 | 0.99908 |
| *MUC2* | 0.75259225 | 1.825978928 | 0.964170953 | 0.33492 |
| *MYF3* | 0.800627605 | 0.608460088 | 0.5483394 | 0.98549 |
| *P15* | 0.247396486 | 1.30510068 | 0.483867859 | 0.11518 |
| *CDKN2A* | 0.871031647 | 0.776504458 | 1.137373696 | 0.76203 |
| *P21* | 0.165502158 | 0.301766191 | 0.435321115 | 0.26204 |
| *P27* | 0.008431373 | 0.034564736 | 0.013807431 | 0.15751 |
| *P57* | 0.155865595 | 0.39719502 | 0.698464432 | 0.12934 |
| *P73* | 0.52949887 | 0.753782966 | 0.327207198 | 0.76958 |
| *PAX5* | 0.824502349 | 0.747058199 | 1.004563827 | 0.79589 |
| *PGK* | 0.83652659 | 0.807753647 | 0.670714021 | 0.92158 |
| *PGR* distal  promoter | 1.53770116 | 1.323774854 | 1.549443746 | 0.89212 |
| *PGR* proximal  promoter | 0.207273074 | 0.542854645 | 0.852742559 | 0.13932 |
| *RAR* | 1.036883889 | 0.942324243 | 0.46654182 | 0.98526 |
| *RASS* | 0.376932035 | 0.391199723 | 0.405119522 | 0.81023 |
| *RB1* | 0.110397057 | 0.368027349 | 0.373626668 | 0.14521 |
| *RFC* | 0.279478226 | 0.473465941 | 0.469236782 | 0.42818 |
| *RIZ* | 0.001051752 | 0.022260214 | 0.01596119 | 0.05303 |
| *RPL15* | 0.20490014 | 0.53736955 | 0.442770473 | 0.23873 |
| *S100* | 0.464719567 | 0.82887768 | 0.552095223 | 0.50464 |
| *SOCS* | 0.577404751 | 0.485480465 | 0.603821053 | 0.88427 |
| *PRKCDBP* | 1.860296813 | 1.743155958 | 1.307847639 | 0.95073 |
| *SYK* | 0.538047068 | 0.505039795 | 0.529641505 | 0.87655 |
| *TES* | 0.396361055 | 0.492055489 | 0.593899408 | 0.58632 |
| *THBS* | 1.058084459 | 0.961479425 | 0.654842813 | 0.97009 |
| *RANKL* | 1.098289796 | 0.983144287 | 1.359217211 | 0.79086 |
| *UPA* | 0.088081451 | 0.508325592 | 0.726045499 | 0.07048 |
| *VHL* | 0.039758037 | 0.781495337 | 0.809670488 | 0.05258 |
| *TMS* | 0.519724136 | 0.882275211 | 0.730710401 | 0.48525 |

Individual, high-prognostic power genes are highlighted in gray.

**Table S4. Pathway analysis.**

| **Canonical Pathway** | **p-value** | **Selected genes** |
| --- | --- | --- |
| p53 Signaling | 0.0000091201083936 | *CDKN2A, CCND2, THBS1, BRCA1* |
| Non-Small Cell Lung Cancer Signaling | 0.0001023292992281 | *CDKN2A, FHIT, RARB* |
| Bladder Cancer Signaling | 0.0001659586907438 | *CDKN2A, DAPK1, THBS1* |
| Tumoricidal Function of Hepatic Natural Killer Cells | 0.0003388441561392 | *ICAM1, CASP8* |
| GADD45 Signaling | 0.0003801893963206 | *CCND2, BRCA1* |
| Ovarian Cancer Signaling | 0.0005754399373372 | *CDKN2A, MSH2, BRCA1* |
| Aryl Hydrocarbon Receptor Signaling | 0.0009332543007970 | *CDKN2A, CCND2, RARB* |
| Molecular Mechanisms of Cancer | 0.0009772372209558 | *CDKN2A, CCND2, CASP8, BRCA1* |
| Retinoic acid Mediated Apoptosis Signaling | 0.0017782794100389 | *RARB, CASP8* |
| Cell Cycle: G2/M DNA Damage Checkpoint Regulation | 0.0019054607179633 | *CDKN2A, BRCA1* |
| Myc Mediated Apoptosis Signaling | 0.0028840315031266 | *CDKN2A, CASP8* |
| Role of BRCA1 in DNA Damage Response | 0.0029512092266664 | *MSH2, BRCA1* |
| Cell Cycle: G1/S Checkpoint Regulation | 0.0038904514499428 | *CDKN2A, CCND2* |
| Small Cell Lung Cancer Signaling | 0.0044668359215096 | *FHIT, RARB* |
| Cyclins and Cell Cycle Regulation | 0.0054954087385763 | *CDKN2A, CCND2* |

P-values are for statistical significance of relationships with known pathways.
