# Supplementary figures and images for: A seven-gene CpG-island methylation panel predicts breast cancer progression
Source: BMC Cancer. 2015 May 19;15:417. doi: 10.1186/s12885-015-1412-9 (PMC4438505; doi:10.1186/s12885-015-1412-9)

## Slide 1
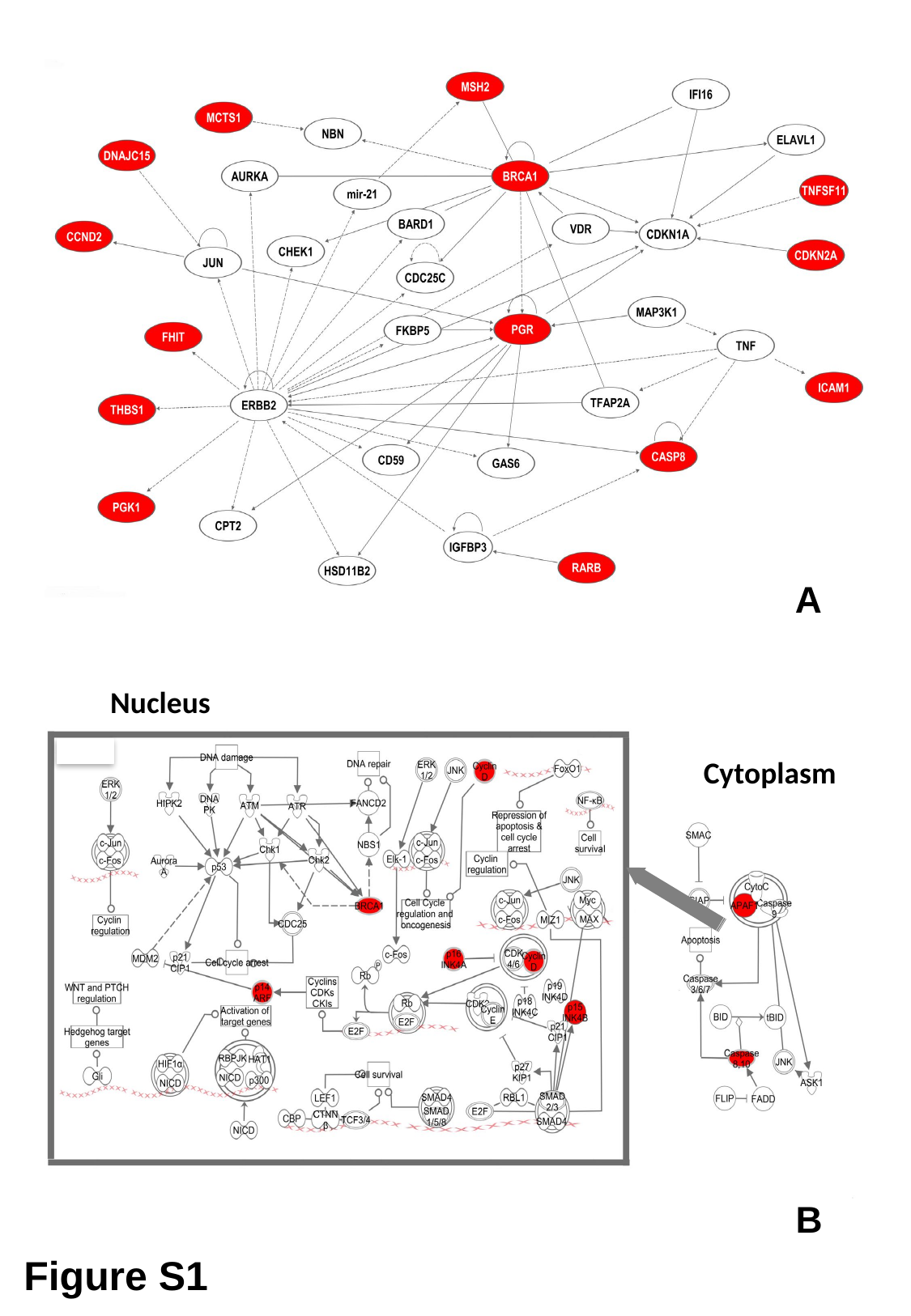

A
Nucleus
Cytoplasm
B
Figure S1

Supplement: Additional file 3: Figure S1. — Gene-protein interaction network - IPA analysis. (A) Network display. Red nodes are directly linked to input genes (Tables 1, 2); white nodes indicate higher iteration/depth. Edges are predicted functional links, and are indicated by arrows. Continuous lines: direct interactions; dashed lines: indirect interactions. (B) Overlay on the Canonical cancer signaling Pathway. Proteins directly linked to input genes (Tables 1, 2) are highlighted in red. Nuclear or cytoplasmic localization is indicated. Relationship with apoptotic pathways is detailed. [file 12885_2015_1412_MOESM3_ESM.pptx]

## Slide 1
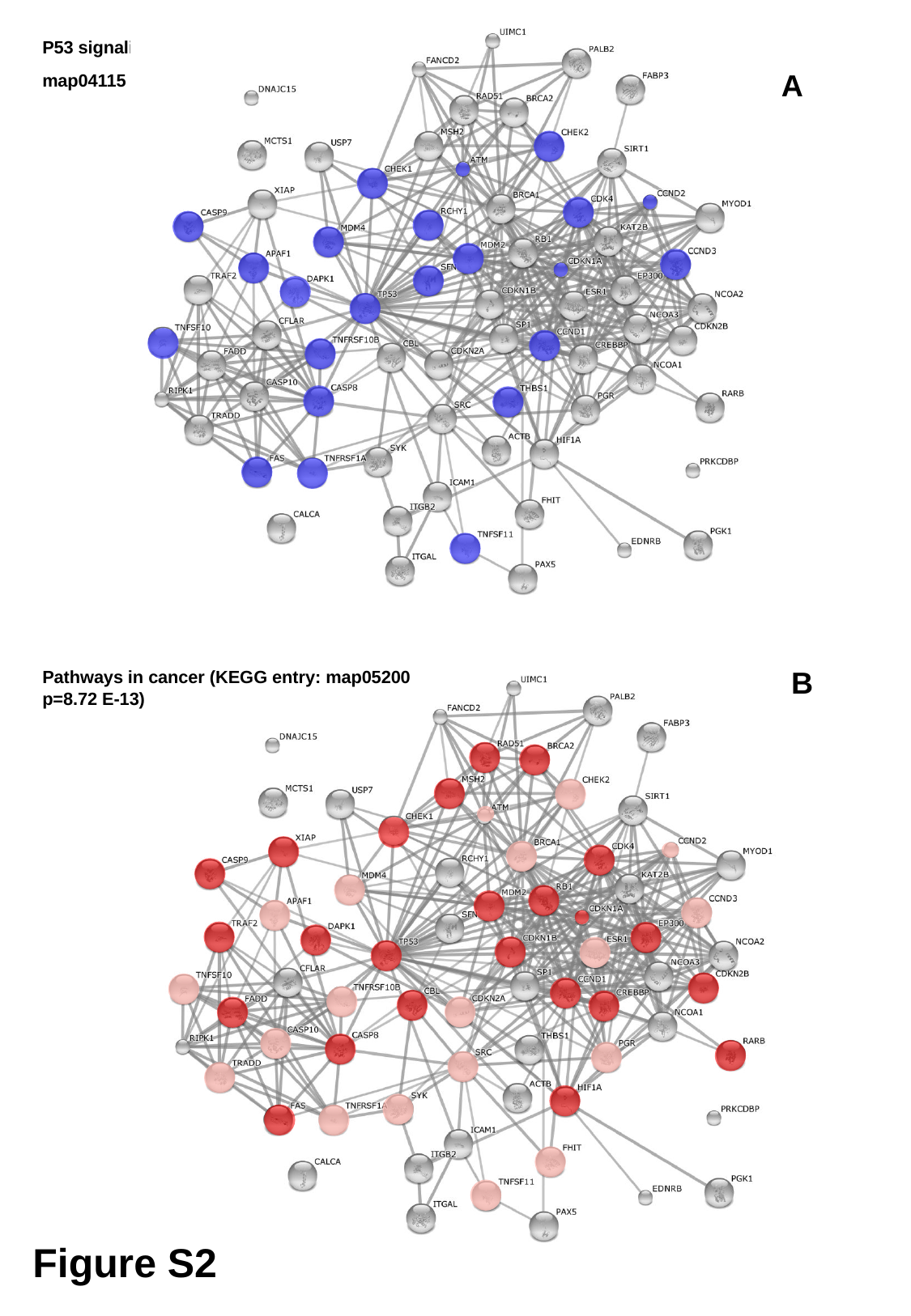

P53 signaling pathway (KEGG entry: map04115 p=6.17 E-22)
A
B
Pathways in cancer (KEGG entry: map05200 p=8.72 E-13)
Figure S2

Supplement: Additional file 4: Figure S2. — Gene-protein interaction network - STRING analysis. Networks including input proteins and protein bridges. (A) p53 signaling pathway (KEGG entry: map04115 p = 6.17 E−22) proteins are highlighted in blue. (B) Pathways in cancer (KEGG entry: map05200 p = 8.72 E−13) proteins are highlighted in red; additional cancer-relevant proteins are highlighted in pink. [file 12885_2015_1412_MOESM4_ESM.pptx]
